# Health seeking behaviour, delayed presentation and its impact among oral cancer patients in Pakistan: a retrospective qualitative study

**DOI:** 10.1186/s12913-019-4521-3

**Published:** 2019-10-21

**Authors:** Sarah Basharat, Babar Tasneen Shaikh, Haroon Ur Rashid, Mamoon Rashid

**Affiliations:** 1 Health Services Academy, Chak Shahzad, Park Road, Islamabad, 44000 Pakistan; 2 Shifa International Hospital, Islamabad, Pakistan

**Keywords:** Delay, Care seeking, Health seeking behaviour, Oral cancer, Public health

## Abstract

**Background:**

Delayed diagnosis of Oral Cancer (OC) can mean a difference in quality and expectancy of life for the patient. This delay could be from the healthcare side, or more importantly from the patient’s side. Globally, there are studies enumerating the causes for delays from the patients’ side in seeking healthcare for Oral Cancer; however, no similar research is found in the context of Pakistan. This study endeavoured to understand the health seeking behaviour, reasons for delay in consultation and the impact on OC patients’ lives.

**Methods:**

In-depth interviews were conducted with randomly selected OC patients at a private sector tertiary care facility in Islamabad (who met the inclusion criteria of having successfully been treated for Oral Cancer) which caters to the most diverse population for the treatment of Oral Cancer. Theoretical saturation was achieved at 14 interviews. All participants gave verbal consent for participation, which was recorded prior to the interviews.

**Results:**

Patients (age range 43–68 years) had received the surgical treatment and radiation. The reported delay before seeking a proper medical advice ranged from 1 month to 2 years. Lack of awareness about OC risk factors, symptoms, and whom to approach for treatment were the main reasons. Most respondents relied on self-treatment considering the non-healing wound/ulcer to be a minor issue until they were advised a consultation with a specialist. Treatment started within 1–3 months after a confirmed diagnosis on biopsy. The reported average expenditure on treatment was US$5000-10,000, mostly covered through a private health insurance and others borrowed the money.

**Conclusion:**

A socio-behavioural change campaign for the general population can result in earlier presentation of the OC, minimizing the financial burden on the patient as well as the health system, and improving the quality of life of the patients.

## Background

Oral Cancer has been documented as a major global Public Health problem [1], and unlike a pattern observed in other cancers, it’s incidence has been reportedly increasing [2]. The WHO lists Cancer as a prominent cause of death globally [3, 4], and nearly 3% of all the cancers in the world are classified as oral cancer [5]. Furthermore, cancer of the oral cavity has reportedly one of the lowest survival rates, in spite of the developments in its management and treatment [6, 7]. In addition, even those who survive may have significant morbidity, residual deformity and reduced quality of life. In spite of the ease of access to the oral cavity, research demonstrates that nearly 50% of the malignant lesions in the oral cavity go unnoticed until they have progressed to an advanced stage [8]. Biomedical research has made advancements in terms of cancer treatment, however, analysing and determining psychosocial and structural factors impacting Oral Cancer are a requirement today [9]. Psychosocial factors have been mentioned as the “psychological characteristics of a person, the social structures, and the interactions between the two which in turn influence the individual’s health behaviours and outcomes” [10].

A timely diagnosis for an oral cancer patient could essentially mean the difference between life and death. The more delayed the diagnosis of oral cancer, the worse the prognosis and hence smaller the chances of survival [6]. The chances of survival in advanced OC can come at a considerable cost which may make it impossible for many patients. There is no agreement on the time beyond which a diagnosis of cancer can be considered to have been delayed [6, 7], however, a delay of one month is said to possibly end up with the patient reaching a more advanced stage of cancer [11] According to a study in Pakistan, nearly 75% of the oral cancer patients first present when the cancer is at T3/T4 level [12], which is strongly indicative of what is known as “Patient Delay” - the delay between the time the patient first notices a symptom to the time s/he has the first consultation [6]. It has been established that knowledge alone does not guarantee action [13], hence raising awareness about risk factors of Oral Cancer although essential, will not be enough as a stand-alone initiative to reduce the burden of the disease.

Moreover, the financial burden incurred during cancer treatment in Pakistan has been documented in some studies [14]. It was discovered that the average expenditure on treatment was more than the patients’ average income. It is important to note here that patients in this study were from a higher socio-economic class of the society and were able to afford the treatment. However, cancer is unfortunately not a disease that differentiates between the poor and the rich.

Hence, the purpose of this research was to understand the health seeking behaviour of Oral Cancer patients in order to better understand the factors that may influence a timely presentation and save lives.

## Methods

This was a retrospective qualitative study comprising in-depth interviews (IDIs) of randomly selected oral cancer patients. The inclusion criteria limited the recruitment to those who had undergone complete treatment and were now cancer free. Shifa International hospital (private) in Islamabad was selected for sample recruitment as in terms of ethnicity and age, it reportedly caters to the most diverse population. The interviews were conducted face to face as well as on phone by the principal researcher - a doctoral candidate having ample experience in qualitative research. Theoretical saturation was achieved at 14 interviews at which point the process of recruitment was stopped.

### Tool development

The IDI discussion guide was developed around the study objectives; to assess the patients’ health seeking behaviour, knowledge about oral cancer, duration of treatment and the total expenditure incurred. It also consisted of demographic factors such as age and gender. The guide was piloted on two recovered OC patients and the wordings of the guide were amended accordingly.

All the patients received surgical as well as adjuvant (Radiation) treatment for their disease. The age range of the interviewed patients was 43 to 68 with four of the respondents being females. The inclusion criteria for the study were; a history of oral cancer, having undergone treatment for oral cancer; currently cancer free; consent to participate. Data collection was continued until theoretical saturation (Additional file 1).

### Patient recruitment

In the absence of valid documented information, it was ascertained through personal communication that the majority of the head and neck cancer patients from Punjab and Khyber Pakhtunkhwa were being treated at Shifa International Hospital. The surgical part of the treatment is the responsibility of a multidisciplinary team which includes Maxillofacial surgeon, Plastic Surgeon, Oncologists, Radiologist and pathologists. The Plastic Surgery department at the Shifa International Hospital takes the lead role in this due to the possibly of gross deformities after surgical resection necessitating reconstruction of the tongue, the jaw or part of the cheek.

The Plastic Surgery department was approached and requested to help with the recruitment of the respondents. One postgraduate trainee, as well as the Head of Department of the relevant unit were both taken on board for the recruitment process, and also for the purpose of keeping the institution involved and in the loop.

Persons at the department then retrieved the files of the patients that matched the inclusion criteria provided to them, and the patients were contacted by the department to obtain their verbal consent to participate in the study. The verbal consent from all participants were also recorded at the start of all interviews. The objectives of the study were explained to all prospective study participants. There were no refusals in the study while the process of recruitment and interviews continued.

### Data collection

After having retrieved the records of patients who fit the inclusion criteria and having approached them to obtain verbal consent to participate in the study, the patients were formally interviewed. Most of the interviews had to be conducted over the phone. Personal meetings with all of the patients for IDIs was a challenge on account of the patients residing in different provinces of Pakistan. Audio recordings of the interviews were made with the consent of the respondents, along with handwritten notes to facilitate data analysis. Each interview lasted for approximately half an hour.

### Data analysis

In the analysis, data is scrutinized, charted and sorted according to key issues and themes using five steps: transcription, familiarization, coding, identifying a thematic framework; and interpretation. Framework analysis provides a brilliant tool to assess procedures and policies from the perspective of the very people who are affected by them [15]. Framework analysis was hence used as the most suited data analysis methodology for this study. No software was employed for the purpose of analysis. For manual analysis, the following steps were undertaken:

1. Transcription

All the interviews and discussions were transcribed. The researchers were interested in the content shared by the respondents, rather than the structure of their responses. Therefore, only significant interruptions (by other members for example), long pauses, and nonverbal communication (such as laughter) were noted. All transcripts were examined for errors by simultaneously listening to the recordings and going through the transcripts. The researchers supplemented the transcripts with notes taken during and immediately after the interviews and the discussions such as for views were given after the recording was stopped and background information essential to the assessment of the content shared.

2. Familiarization

The researcher then scrupulously read and re-read each transcript, along with listening back to the recordings in order to become familiar with the data set. The initial impressions were recorded in the margins of the transcripts, for example where participants expressed exceptionally strong or two contrasting views. Familiarization through reading and making notes in this way also enabled the researcher to navigate through the transcripts with ease.

3. Coding

For the purpose of coding, two margins were drawn on either side of the page. The principal researcher then proceeded to underline interesting segments of text and used the left-hand margin to describe the content of each passage with a label or code. This ranged from a few words to whole paragraphs. The right-hand margin was then used to record more detailed notes and ideas such as questions to bear in mind as the analysis proceeded, and ideas for explanations or patterns in the data.

4. Analytical Framework

The thematic areas which were to be explored were pre-defined (also known as a priori themes). When preparing for the study, it was decided that three thematic areas need to be covered to address the issue at hand: Awareness on the patient’s side pertaining to Oral Cancer risk factors and management (to assess how these factors contributed to Action) and the actions they took as a result of their awareness; Delay from the patient’s side as well as factors from the healthcare delivery side which hampered timely acquisition of care; and Impact of the culminating treatment plan in their particular case.

Following the above process, an analytical framework was developed to identify which codes and their corresponding segments from the interviews fell under which theme. Refining the analytical framework was an iterative process which was repeated until no new codes were generated. The final framework consisted of five (5) codes.

The final analytical framework appeared as shown in Table 1.

## Results

### Knowledge about Oral Cancer

The main reported symptom of cancer was a chronic non-healing wound (e.g. following tooth extraction) and/or a spontaneously appearing non-healing ulcer. Most of the respondents considered the non-healing wound/ulcer to be some minor issue until they mentioned it to someone and were advised to seek medical help. Even though the level of knowledge regarding the symptoms of OC was found to be low (i.e. none of the patients found their lesions to be suspicious, and none could have imagined it could turn out to be cancer), the majority of the patients at the time of the interviews were nevertheless aware of the factors that cause or may cause OC, including consumptions of products that lead to the irritation of the oral mucosa, such as betel quid.

*“Yes. Some people use paan (betel quid), some smoke, sometimes people don’t take care of their oral hygiene because in Pakistan we don’t know much about that. These habits lead to Oral Cancer. We see warnings and pictures on cigarette boxes.” - 43-year-old recovered male OC patient*

Apart from oral hygiene being frequently reported as a potential cause of Oral Cancer, spicy food was also seen as a risk since it irritates the oral mucosa.

*“She ate gol gappay (a spicy street food) a few times after which her tongue got affected and she told me about it.” - Son of 65-yer-old recovered female OC patient*

Nevertheless, some of the patients who did not have a history of tobacco consumption, and had developed cancer, believed that the divine force played a role. The patients reporting divine intervention were unable to give a reason for being subjected to the misery. Religion being a sensitive topic of discussion, this issue was not further probed lest it be perceived as a question on their faith.

*“We hear about all that, and it is the people who smoke that get cancer but my wife doesn’t smoke. It just happened to her. It’s God’s will. He tests us in these ways.” - Husband of 52-year-old recovered female OC patient*

Here the meaning of “test” was meant as a test of your faith and fortitude with which you bear adversity. It is common belief in Pakistan that if you put up with adversity with grace and fortitude, you will eventually be rewarded for your patience.

This issue was not probed further.

### First point of contact

The first person(s) most patients approached were family and friends, who then suggested the patient see a healthcare professional. Once they sought medical help, things progressed quickly with the patients being swiftly referred from one doctor to another until they reached the consultant.

*“* *I told my son* *about an ulcer on my tongue that had been there for a while.” - 68-year-old recovered male OC patient*

Another important observation was the implied dependency by females on males for action. This is not surprising and in line with the established cultural norms of the region. Females are as a rule not free to move around and require a male to help them navigate the healthcare system. In most cases women are dependent on males for movement from one place to another (i.e. they can either not drive, or are reluctant to leave home alone without a guardian).

The quote in the previous section was the son of the elderly lady who was diagnosed with a malignant lesion on her tongue. The sun was her first point of contact when he shared that:

*“She ate gol gappay (a spicy street food) a few times after which her tongue got affected and she told me about it.” - Son of 65-yer-old recovered female OC patient*

In this particular case, the son was the first point of contact for the female, and she remained dependent on him for her subsequent visits. It can thus safely be assumed that any delay from the son in seeking medical help, would have played a major role in the delay from the patient’s side for seeking help.

*“Yes, I was the first person she told. I then took her to the doctor.” - Son of 65-yer-old recovered female OC patient*

### Delays in health care seeking

There was a delay of nearly one month to two years for seeking help. Lack of awareness about oral cancer risk factors, prognosis, symptoms, and about the right doctor to approach for treatment were the main causes for the delay.

*“After he developed blisters and ulcers on his tongue they weren’t getting healed. For nearly four to five years we kept visiting a doctor who said it was a problem with his stomach and acidity.”- Daughter of 53-year-old male OC patient*

In this case, since the patient was not reluctant to seek help, a lack of awareness regarding suspicious lesions on part of the patient’s daughter (who was the main point of contact to approach the patient), as well as the patient himself, played a vital role.

The biopsy results on average took one to two weeks and the treatment started within one to three months from the first time the patient sought help. All the patients were able to have their first consultation on the day they attempted to approached the consultant and on average the delay between a confirmed diagnosis and surgery was one week or less.

### Expenditure

The expenditure reportedly incurred on treatment ranged from PKR 600,000–1200,000 (USD 5000-10,000). However, most of the respondents said they did not find it difficult to arrange the money as most reported using some form of private insurance. Nevertheless, other expenses related to visits of the hospital were quite cumbersome. Those who had no insurance also reportedly found it an uphill battle to arrange the required amount. Two respondents claimed finding it impossible to afford the treatment and had to take a loan.

*“It was very difficult for us. We spent everything we had on the treatment.”- 68-year-old recovered male OC patient*

*“The treatment was very expensive, but the disease was worse. It was painful, my face was disfigured.” - 43-year-old recovered male OC patient*

### Impact of delayed health care seeking

Besides serious financial implications on the family, most of the respondents felt that oral cancer had a very negative impact on their personal and professional lives, most commonly due to pain. However, once they were surgically treated they believed that the quality of their lives had returned nearly to what it was before they got ill.

*“The treatment was expensive, but the disease was worse. It was painful, my face was disfigured. But see now I can talk, eat, and I can live my life [because of the successful treatment which included surgical repair].” - 43-year-old recovered male OC patient*

*“The disease very much affected her life. Over time it became difficult for her to talk or eat. Of course her life was very negatively affected. There is now an immense improvement with the treatment” - Husband of 52-year-old recovered female OC patient.*

**Fig. 1:**
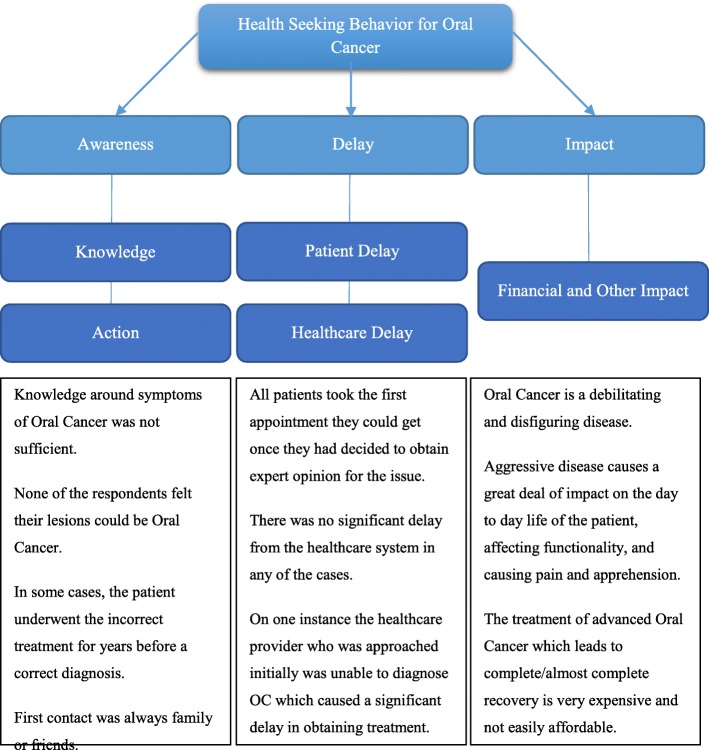
Summary of main findings under various themes

Figure 1 provides a summary of the main study findings in light of the themes and codes generated.

**Table 1:** Analytical Framework for the Analysis of the Interviews

Theme	Code	Description
Awareness	Knowledge	Knowledge about risk factors for oral experience
	Action	When the patient decided to seek help, whom they approached first, what prompted them to seek help
Delay	Patient Delay	How long after discovering the lesion did the respondent decide to seek help, how long after deciding did they approach a healthcare provider
	Treatment/ Healthcare Delay	How long did it take for a definite diagnosis to be reached What was the suggested treatment, how long did it take from the time of the diagnosis for the treatment to be started
Impact	Financial and other Impact	Cost of treatment, how was the money arranged, how big a challenge was it to arrange the money, impact of their experience on their quality of life.

## Discussion

This study was concerned with the process of Oral Cancer discovery, management, treatment, and recovery. As such, only those patients were included who had undergone treatment successfully. The inclusion criteria limited our recruitment to a particular socio-economic class, which in turn further helped to highlight the financial burden the patient and his/her family needs to bear, making treatment an often insurmountable hurdle for the lower socio-economic classes. Nevertheless, the findings have led us to draw certain inferences, based on which a comprehensive strategy will be briefly discussed here, which will account for the socio-economic gaps in the society we’re aiming to protect against the detrimental impacts of Oral Cancer.

In general, an early diagnosis leading to receiving early treatment is the pivotal point in the management of cancer patients. This is even more applicable to OC patients since the potentially malignant lesion remains localized, without healing, for a long time and if the patient presents when the disease is at Stage 1, a cure rate of above 90% can be achieved [16]. There are three major facts concerning OC. Firstly, it is preventable in the majority of patients. Primary prevention is possible by community education about OC symptoms and by decreasing the use of tobacco through awareness raising and legislation. Secondly, OC is preceded by a pre-cancerous lesion which is usually non-healing and is an early warning sign. Thirdly, if a lesion develops into OC, it is curable with a high success rate if it is detected at an early stage. The majority of the patients presented when the disease had reached an advanced stage, which is consistent with the findings of other studies in Pakistan identifying delayed presentation as a prevalent problem on part of the patients in this region [15, 17]. There have been a number of studies on the determinants of delay in presentation of cancer patients in general [18], in malignant melanoma [19], and in breast cancer [20]. These studies validate our findings where most of the patients presented nearly one month to two years after discovering the lesion.

It is proven that masses must be educated on the urgency for an early consultation for patients with suspected oral lesions [21, 22]. This study has demonstrated a low level of knowledge around the symptoms of oral cancer, and also a tendency to attribute disease occurrence to divine will. This finding is consistent with those reported in another qualitative study, in which one of the major factors identified as being responsible for delay in presentation of oral cancer for more than 6 months was ignorance; while belief in destiny was also identified as a minor factor [23, 24]. Hence any intervention aimed at altering an OC patient’s health seeking behaviour must factor in this tendency to accept fate, destiny and divine will as the causative factors for OC.

It has been established that knowledge alone does not guarantee action [13], hence raising awareness about risk factors of Oral Cancer although essential, will not be enough as a stand-alone initiative to reduce the burden of the disease. The reasons could be manifold, but in the light of the current study it became apparent that a lack of understanding regarding the symptoms of Oral Cancer, and confusion around deciding to seek medical help are the main contributing factors in the delays from the patients’ side. The idea of cancer is scary, but fear of diagnosis did not appear to the be one of the deciding factors in the delays caused.

To address the said issues, educating the healthcare providers especially regarding identification and differential diagnoses of non-healing lesions inside the oral cavity, along with educating the community regarding the presentation of suspicious oral lesions that could be symptomatic of malignancy, the risk factors for oral cancer, the importance of an early diagnosis, and instructions regarding seeking help would allow them to be conscious of their health and could possibly encourage a timely action.

More elaborately, a system put in place for screening of high-risk populations, and identification and timely referral of suspicious lesions would be required. Opportunities need to be provided to people who actively seek help, and the system needs to prepared to receive and refer them efficiently for a timely management. Such a system needs to be set up as a multi-pronged strategy along all strata of the healthcare system, ranging from policy and budgetary issues, to training and monitoring of the healthcare staff.

**Fig. 2:**
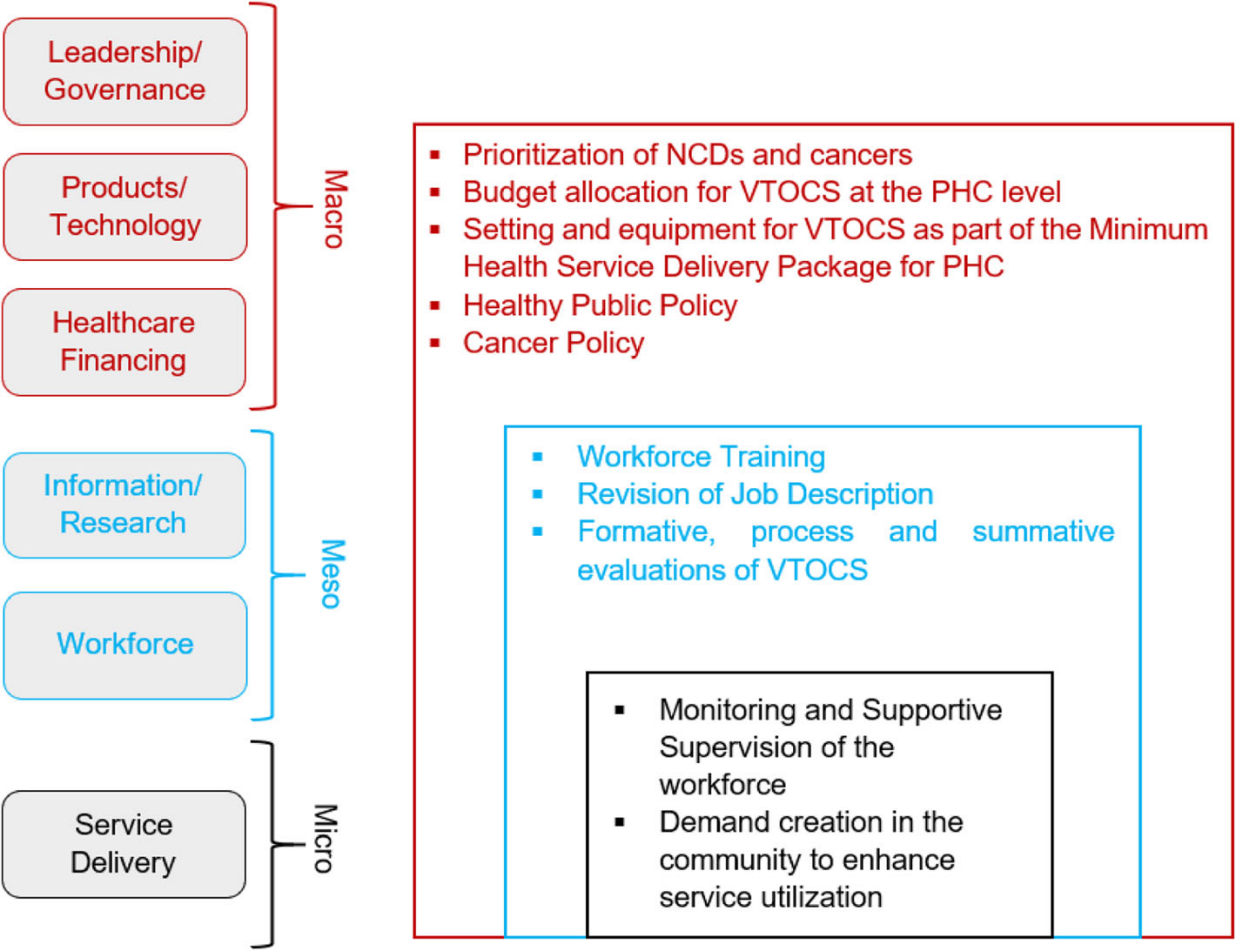
Strategy for Early Diagnosis and Timely Management of Oral Cancer

Figure 2 indicates the devised strategy for the way forward to ensuring a timely diagnosis, referral, and treatment of Oral Cancer patients as designed around the six building blocks of the healthcare system as identified by the World Health Organization.

### Research transferability, dependability, confirmability, and credibility

#### Credibility

In case of qualitative research, credibility can only be judged by the study participants, since we are asking them about their own experiences, perceptions, and views. We have assumed that the information shared by the participants is accurate, and hence the information that we have acquired, is credible. Involving a third party for recruitment and to help with the interviews further helped with the credibility by ensuring that the researchers’ internal biases did not influence the process of selection nor of interviewing.

#### Transferability

In case of this research, the sample was randomly selected from among a list of patients meeting the inclusion criteria. Interviews continued until no new information could be retrieved. Moreover, the differences in the respondents’ ethnic backgrounds were not accounted for, nor were the results of the study factoring in the local ethnic factors contributing to the delays. The socio-cultural factors which emerged and played some role in the patient delay, are not new and are prevalent across the country. Moreover, since the inclusion criteria narrowed down the selection to those who had received treatment for and recovered fully from Oral Cancer (to be able to shed light on the financial burden of the treatment, how easy/difficult it was for the patients to manage, and the actual impact of the disease along with their journey to recovery), the lower socio-economic class of the society that goes undiagnosed and untreated, was not part of the study. Hence all of the results cannot hold true for all Oral Cancers patients from all socio-economic strata of the society. However, for all Oral Cancer patients in Pakistan undergoing treatment, the results we believe are transferable by virtue of the planning put into place prior to the start of the study, and the objectives of the study.

#### Dependability

Since the guide was piloted and modified according to the results from the pilot, we believe if the methodology is followed to the t, similar results may be reproduced in any setting within Pakistan.

#### Confirmability

The interviews were individually recorded, and the information provided was confirmed, and reconfirmed not only during the interviews but also during the analysis to ensure there were no discrepancies. Most of our findings were confirmed by other existing and published research. This is further discussed in the discussion section, where we have provided appropriate references where we have attempted to compare the collected information with what is published.

## Conclusion

In order to address the preventable problem of a delay in OC presenting at a health facility, a comprehensive socio-behavioural communication strategy is required, tailor made for OC patients, health care providers as well as the general population, explaining about the risk factors, symptoms and treatment options and complications of the OC, as well as emphasizing the importance of an early check-up and initiation of treatment.

## Supplementary information

**Additional file 1.** In-depth interview guide.

## Data Availability

The datasets generated and/or analysed during the current study are not publicly available because the research conducted is part of a doctoral degree yet to be defended; but are available from the corresponding author on reasonable request.
