# Correction to: Health seeking behaviour, delayed presentation and its impact among oral cancer patients in Pakistan: a retrospective qualitative study

**DOI:** 10.1186/s12913-019-4716-7

**Published:** 2019-11-14

**Authors:** Sarah Basharat, Babar Tasneem Shaikh, Haroon Ur Rashid, Mamoon Rashid

**Affiliations:** 1 Health Services Academy, Chak Shahzad, Park Road, Islamabad, 44000 Pakistan; 2 Shifa International Hospital, Islamabad, Pakistan

**Correction to: BMC Health Serv Res**

**https://doi.org/10.1186/s12913-019-4521-3**

In the original publication of this article [1], an author’s name needs to be revised from Babar Tasneen Shaikh to Babar Tasneem Shaikh.

The original article has been corrected.
