# Supplementary material for: Health seeking behaviour, delayed presentation and its impact among oral cancer patients in Pakistan: a retrospective qualitative study
Source: BMC Health Serv Res. 2019 Oct 21;19:715. doi: 10.1186/s12913-019-4521-3 (PMC6805330; doi:10.1186/s12913-019-4521-3)
Supplement: Supplementary file 1 — Additional file 1. In-depth interview guide. [file 12913_2019_4521_MOESM1_ESM.docx]

**In-depth Interview guide**

| Questions | Probes |
| --- | --- |
| *Information available regarding the stage of cancer at the time of the first consultation?* | *If yes, please mention the stage of cancer* |
| Sex |  |
| Age |  |
| Marital status |  |
| Employment |  |
| Date of first contact with the doctor |  |
| Date of first consultation |  |
| What prompted you to see a doctor for this particular problem? | - Was there pain? - Did someone suggest it? Who? Why? - Was there discomfort? Please elaborate - Any other reason? Please elaborate |
| Whom did you first approach with the problem? | - A family member? - A friend? - A local healthcare provider? (please elaborate - Which health care facility/health care provider? Why did you choose to approach this particular HF? |
| How long did you wait before reaching the decision to seek help for the problem? |  |
| How long did you wait to approach a health care provider for your problem, after you decided to seek help? | - If there was a delay, how long was it? - What was/were the cause/s of the delay? |
| How long after your first consultation did you start receiving treatment? |  |
| What was the treatment that you received? |  |
| How long did the treatment take? |  |
| How much have you approximately spent on your treatment? |  |
| Who paid for it? |  |
| On a scale of 1 to 10, with one being very easy and 10 being very difficult, how easy or difficult would you say it was for you to afford the treatment? |  |
| How has your problem affected your life? | - Are there any activities you could do before and have been unable to do because of the problem? - Has it had an impact on your job/employability? How? - Has it had an impact on your family life/marital life? |
